# Flexible surface acoustic wave technology for enhancing transdermal drug delivery

**DOI:** 10.1007/s13346-024-01682-y

**Published:** 2024-08-06

**Authors:** Jikai Zhang, Duygu Bahar, Hui Ling Ong, Peter Arnold, Meng Zhang, Yunhong Jiang, Ran Tao, Luke Haworth, Xin Yang, Chelsea Brain, Mohammad Rahmati, Hamdi Torun, Qiang Wu, Jingting Luo, Yong-Qing Fu

**Affiliations:** 1 Faculty of Engineering and Environment, Northumbria University, Newcastle Upon Tyne, Newcastle NE1 8ST UK; 2 Hub for Biotechnology in the Built Environment, Department of Applied Sciences, Faculty of Health and Life Sciences, Northumbria University at Newcastle, Newcastle Upon Tyne, NE1 8ST UK; 3 Shenzhen Key Laboratory of Advanced Thin Films and Applications, College of Physics and Energy, Shenzhen University, Shenzhen, 518060 China; 4 Department of Electrical and Electronic Engineering, School of Engineering, Cardiff University, Cardiff, CF24 3AA UK; 5 IP & Commercialisation, Research and Innovation, Northumbria University, Newcastle Upon Tyne, Newcastle NE1 8ST UK

**Keywords:** Surface acoustic wave, Drug delivery, Molecules, Wearable electronics, Transdermal drug delivery

## Abstract

Transdermal drug delivery provides therapeutic benefits over enteric or injection delivery because its transdermal routes provide more consistent concentrations of drug and avoid issues of drugs affecting kidneys and liver functions. Many technologies have been evaluated to enhance drug delivery through the relatively impervious epidermal layer of the skin. However, precise delivery of large hydrophilic molecules is still a great challenge even though microneedles or other energized (such as electrical, thermal, or ultrasonic) patches have been used, which are often difficult to be integrated into small wearable devices. This study developed a flexible surface acoustic wave (SAW) patch platform to facilitate transdermal delivery of macromolecules with fluorescein isothiocyanates up to 2000 kDa. Two surrogates of human skin were used to evaluate SAW based energized devices, i.e., delivering dextran through agarose gels and across stratum corneum of pig skin into the epidermis. Results showed that the 2000 kDa fluorescent molecules have been delivered up to 1.1 mm in agarose gel, and the fluorescent molecules from 4 to 2000 kDa have been delivered up to 100 µm and 25 µm in porcine skin tissue, respectively. Mechanical agitation, localised streaming, and acousto-thermal effect generated on the skin surface were identified as the main mechanisms for promoting drug transdermal transportation, although micro/nanoscale acoustic cavitation induced by SAWs could also have its contribution. SAW enhanced transdermal drug delivery is dependent on the combined effects of wave frequency and intensity, duration of applied acoustic waves, temperature, and drug molecules molecular weights.

**Graphical Abstract:**

Graphical Abstract: Flexible surface acoustic wave technology for enhancing transdermal drug delivery

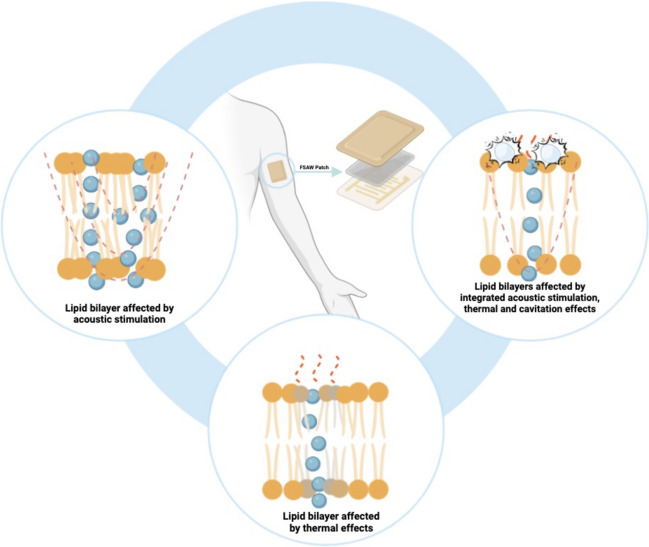

**Supplementary Information:**

The online version contains supplementary material available at 10.1007/s13346-024-01682-y.

## Introduction

Transdermal drug delivery, as an alternative to oral and intravenous subcutaneous injection ones, allows painless delivery of drugs or macromolecules through the skin into the body [1]. Unlike needle injections, transdermal drug delivery method offers minimally invasive delivery [2] in addition to avoidance of drug degradation in the stomach [3], and potentially controls precise release of drugs [4]. In the transdermal drug delivery, drug molecules are required to penetrate layers of epidermis [5], dermis [6] and hypodermis [7] before reaching the blood circulation to exert drug action. This can be challenging especially considering that stratum corneum (SC), the outermost barrier of the skin, is consisted of a 15–20 µm-thick layer of keratin-filled dead cells [8], which is the key barrier to transdermal drug delivery.

Various chemical or physical modalities have been used for transdermal delivery of small molecules of lipophilic drugs, with their molecular weights often less than 500 Da, allowing them to be diffused through the SC to enter the blood stream [9]. Various chemical methods have been reported to enhance skin permeability primarily by disrupting lipid bilayer structure in the SC or by creating lipid accumulation defects, thereby enhancing the diffusion. However, these chemical methods often unavoidably irritate or affect the living cells beneath the SC [10–12]. On the other hand, physical methods such as iontophoresis [13], electroporation [14], microneedles [15] and thermal ablations [16] have also been used for transdermal drug delivery. These physical methods are more suitable for drugs with smaller molecular weights and hydrophobic features. However, for macromolecular drugs or highly hydrophilic drugs, these physical methods often have limitations, mainly because the presence of the lipid layer in the outermost SC of the skin is difficult for hydrophilic molecules to penetrate. Whereas many of these methods cannot easily change the hydrophilic nature of drugs, and therefore they have limited effects on highly hydrophilic drug molecules [17, 18].

Ultrasonic methods (with frequencies generally from tens of kHz up to a few MHz) have been highly regarded for transdermal drug delivery and they mainly rely on the acoustic cavitation effect within the coupling media between the ultrasonic device and skin surface [19–22]. Ultrasonic devices generate high intensities of shock waves onto the lipid layer, thereby enhancing skin penetration. Although being effective for delivery, ultrasound methods used for drug delivery can sometimes cause harm of adjacent healthy tissues, a risk which is severe in sensitive areas such as the brain [22]. Shear forces generated by cavitation of bubbles could cause cell deformation or death [23], or DNA damage [24, 25], or reduction of cell viability [26].

Surface acoustic waves (SAWs) have recently been extensively investigated to manipulate, pattern, and separate biological cells [27] or solid particles [28], and drive the flowing liquid or droplets for streaming, pumping, jetting, or nebulization [29]. SAWs generated by amplified radio frequency (RF) signals were applied to the interdigital transducers (IDTs) on a piezoelectric substrate, thus resulting in laterally and longitudinally propagating waves along the surface [30]. Conventional SAW devices are rigid, as they are often made onto piezoelectric substrates such as lithium niobate, or onto dielectric substrates such as silicon and glass substrates coated with piezoelectric films [31]. This rigidity impedes effective adherence to arbitrarily shaped surfaces, rendering them unsuitable for being integrated into wearable devices such as for skin drug delivery usages. Flexible acoustic wave devices (FSAW), developed on polymer and metallic foil substrates (such as polymer or aluminium foils), are promising for wearable applications attributed to their bendability and good ductility [32]. Zinc oxide (ZnO) and aluminum nitride (AlN) have been successfully demonstrated as piezoelectric thin film layers of FSAWs for wearable biosensors and lab-on-chips [33, 34]. For a FSAW device, a hybrid wave mode consisting of Rayleigh and Lamb waves is often generated and applied for different applications [35, 36].

**Fig. 1:**
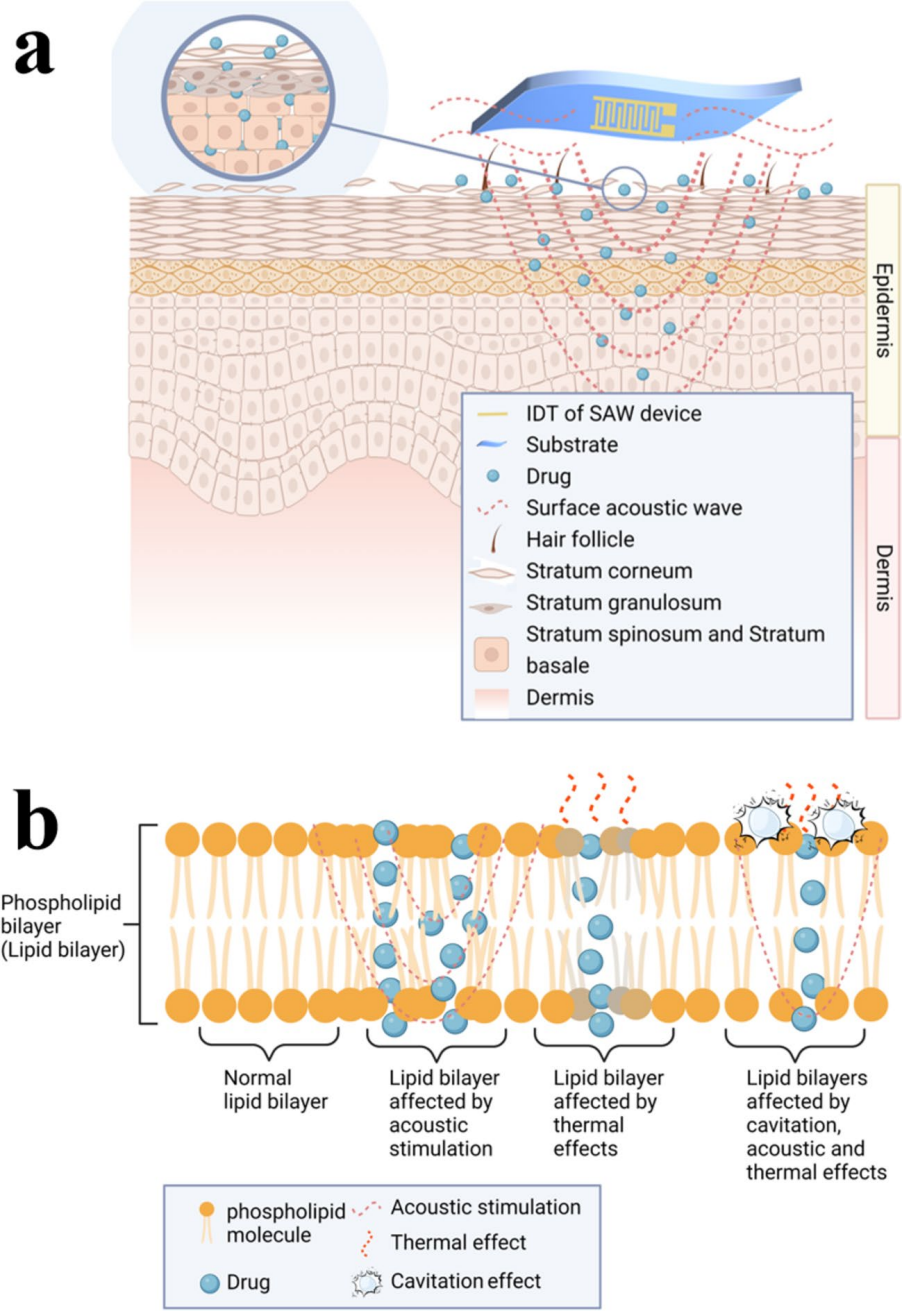
(**a**) Drug delivery mechanisms and route induced in the skin layers using the SAW technique; (**b**) Drug delivery routes in the lipid bilayer using the SAW technique

Similar to the ultrasonic methods, SAW excitation can be used to penetrate the layers of skin for transdermal drug delivery. SAW technology has previously been demonstrated as a potential minimally invasive drug delivery method but without generating cavitation effects due to high frequency devices used [37]. Compared to the traditional chemical or ultrasonic transdermal drug delivery, SAWs mainly affect the epidermal layer, thus avoiding the damage of living cells located in the deeper layers of the skin, reducing irreversible damage during transdermal delivery. The acoustic waves generated by the SAW device can transmit perpendicularly to the skin surface, driving the drug molecules to diffuse laterally and penetrate longitudinally into the intercellular lipids, breaking through the first layer of the skin barrier, as illustrated in Fig. 1. When the SAWs are applied on the skin surface, nanoscale and high-frequency mechanical vibrations promote the diffusion of drug molecules on the skin surface. They can also interfere and deform the lipidic structures on the SC by generating localised pressure and heating effects, which can effectively promote the delivery process.

Figure 1a summarises the proposed drug delivery mechanisms and various routes induced in the skin layers using the SAW technique. For SAW activation methods, transdermal administration follows two common routes, i.e., trans-epidermal and trans-follicular routes [38]. During SAW agitation, the nanoscale earthquake effect causes the significant localized vibrations and radiation acoustic waves, causing the disrupted SC layers (see Fig. 1b). Acoustic induced heating effect or thermal energy generated by the SAWs due to significant energy dissipation promotes the diffusion of drug molecules [39] (see Fig. 1b). In the trans-epidermal delivery pathways, applications of low frequency (down to a few MHz) SAWs usually induce the nucleation and oscillation of microbubbles. Upon collapsing, these bubbles could alter the structure of the SC’s lipid layer, thereby creating channels that facilitate diffusion by forming the passages of drug molecules through the SC.

Hydrophobic drugs traverse the cells aided by the lipid layer, while hydrophilic drugs penetrate the SC layer due to the keratinocytes' hydrophilic nature, ultimately reaching the systemic circulation [38, 39]. Trans-follicular penetration is also important for the transdermal delivery of macromolecular drugs [40], and under the strong agitation of SAWs, drug molecules could pass through the SC through hair follicles and sweat glands and enter the systemic circulation.

However, currently there are not any previous studies to explore the efficacy of flexible SAW (FSAW) devices for the controlled transdermal drug delivery, especially for large macromolecular compounds. Therefore, in this study, it is aimed to develop flexible SAW patch platform based on a thin aluminum sheet (with a thickness of 200 µm) for effective transdermal drug delivery. This is the first demonstration of delivering large macromolecular compounds with average sizes ranging from 4 to 2000 kDa using the FSAW technology, through skin simulant agarose gels and pig skin (including pig ear or pig belly). The efficacy of the FSAW device for the controlled transdermal delivery of various macromolecular drugs were investigated.

## Experimental

### Preparation of SAW device and skin and drug models.

#### Flexible ZnO/Al SAW devices were used in this study and their fabrication process is described in the supporting document “S1. SAW device fabrication.”

For the preparation of skin model, agarose powders (Melford, UK, with its gel strength of 1200 g/cm^2^) were dissolved in deionized water and mixed thoroughly under a continuously stirring process. The obtained solution was heated in a microwave (with a power of 700 W) for one minute to fully resolve the agarose powder. The obtained liquid was poured into a Petri dish to obtain a layer with a thickness of 0.3 mm – 0.6 mm for testing. Agarose gels with different concentrations were used as a scaffold to mimic the skin layer (i.e., with concentrations of 10% and 20%).

The samples of pig belly and pig ear were used as a skin model for application demonstrations. The structure of pig ears is similar to that of human skin, consisting of epidermis, dermis and subcutaneous fat layers, which makes it suitable for simulating human skin [41–43]. Fresh pig belly and pig ears, provided by Wrefords’ Farm, Newcastle upon Tyne, UK, were stored in dry ice, and brought back to the laboratory for testing within 2 h. The pig ear was initially cleaned with DI water, followed by excision of the dorsal skin from the underlying cartilage using a scalpel. The subcutaneous fat tissue was removed using surgical scissors to obtain a full-thickness skin (500∼800 μm), which was then washed with distilled water and visually inspected to ensure its integrity.

Fluorescein isothiocyanate-dextran (FITC) molecules (Sigma-Aldrich) with their molecular weights of 4 kDa,10 kDa, 40 kDa, and 2000 kDa were mixed into 1 ml of deionized water to prepare a test solution of 10 mg/ml, respectively.

### SAW enhanced drug delivery using cryostat methods

#### Experimental setup

A signal generator (AFG1062, Tektronix) was used to generate a radio frequency (RF) signal. Amplified by a power amplifier (75A250, Amplifier Research), the signals were input to the IDTs of the SAW device to generate SAWs. The setup is shown in Figure S1a in the supporting information. A power meter (9104, Racal Dana) was used to measure the power input to the IDTs of SAW device. The surface temperature of the SAW device was measured using a thermocouple (2029 T, Digitron).

#### Cryosection operation procedures

The agarose gel was prepared into a square shape with a uniform thickness at given thickness between 0.3 mm to 0.6 mm and the measured dimension was ~ 1.5 × 2 cm^2^. The backside of the SAW device (opposite to the IDT side) was gently pressed onto the gel surface, fixed with metal pins to the pads of the SAW device, so that the FITC paste of 0.1–0.5 ml was applied to the agarose. During the tests, the power outputs of the RF signals were between 0.002 W and 5.400 W, respectively. After the test, the sample with the remaining FITC on the surface was thoroughly cleaned using a fiber-free soft cloth and then washed with running phosphate-buffered saline (PBS, Sigma-Aldrich) solution for 60 s. The central location in close contact with the IDT region was selected for longitudinal sectioning, with a slice thickness of ~ 0.1 mm. The tested samples were fixed using the optimal cutting temperature (OCT, Scigen) compound after cleaning and sliced into sample thickness of ~ 30 μm using a low-temperature cryostat (Model OTF/A5) at -30℃. The drug delivered pig skin was also cut into a uniform size (1.5 × 2 cm^2^) using the same test bench and test procedures as in the agarose gel test listed above.

#### Drug delivery depth analysis using fluorescence signals

Measurements of fluorescence signals for both agarose gel and pig skin experiments were performed using a fluorescence microscope (DM5000 B, Leica) with a laser wavelength of 488 nm under 5X and 10X magnifications. As it is well-known, drug transdermal diffusion is regarded as the process of dynamic diffusion of a drug from high concentration to low concentration, which can be simplified into one-dimensional diffusion case within a plane based on the Fick’s Second Law:

1 $$\frac{\partial c(x,t)}{\partial t}=D\frac{{\partial }^{2}c}{\partial {x}^{2}}$$

where *x* and *t* are the length and time coordinates, *c* is the concentration of the diffusing species, *D* is the diffusion coefficient. Under the critical conditions, c (x = 0) = ${c}_{s}$. The value of concentration c is a fixed constant ${c}_{s}$; c_(x = ∞)_ = ${c}_{0}$, corresponding to the original concentration of chemical existing in the phase, c_0_ remains constant in the far bulk phase at $x\hspace{0.17em}=\hspace{0.17em}\infty$.

2 $$\text{C}\left(\text{x},\text{t}\right)={\text{c}}_{s}- ({c}_{s}- {c}_{0})\text{erf}\frac{\text{x}}{2\sqrt{Dt}}$$

After obtaining the fluorescent images of the slices, the fluorescent signal distribution was analyzed using MATLAB and Image J software, and then used curve fitting Eq. (2) to obtain the transport distances.

### SAW enhanced drug delivery using Franz cell

#### Experimental setup

Franz cell based drug quantification method was applied to evaluate the delivery efficiency. In this work, the permeation of the drug molecules in pig ear and agarose gel was studied in vitro using a Franz cell module (4G-02–00-30–20, PermeGear) quantitatively. The setup is shown in Figure S1b in the supporting information. The receptor chamber was filled with 20 mL PBS solution. The pig ear (with a thickness in a range from 0.5 mm to 1 mm) or agarose gel (with a thickness in a range from 0.3 mm to 0.5 mm) was placed over the flat ground of the receptor chamber and held by the metal clamp of the Franz cell module. The experimental setups utilized 4, 10, 40 and 2000 kDa FITC molecules at concentrations of 1–6 mg/mL and a volume of 0.1–0.5 mL, coupled with a 13.51 MHz FSAW device. After the FITC solution was applied on the surface of pig ear skin and agarose gel, the SAW device was put on top of surface and different SAW voltages/powers were applied for different durations to drive the FITC passing through the layer into the liquid of receptor chamber.

#### Absorbance analysis

The PBS solution after the experiment was collected, and an ultraviolet–visible (UV–VIS) spectroscope (UV-2600, Shimadzu) was used to measure the absorbance at 495 nm. Then, the concentration and diffusion ratio of the drug were obtained by comparing and calculating based on the FITC standard and calibrated solution curve, as shown in Figures S2 and S3 in the supporting information. At least three repeated samples were tested, and average values were obtained. Here the transportation capacity of drug delivery is defined as:

3 $$\text{t }=\frac{{m}_{d}}{{m}_{t}}\times 100\%$$

where t is the transportation ratio, ${m}_{d}$ is the transport mass, and ${m}_{t}$ is the total mass in the experiment.

## Results and discussions

### SAW induced delivery results into Agarose gels

#### FITC molecular weight effect

**Fig. 2:**
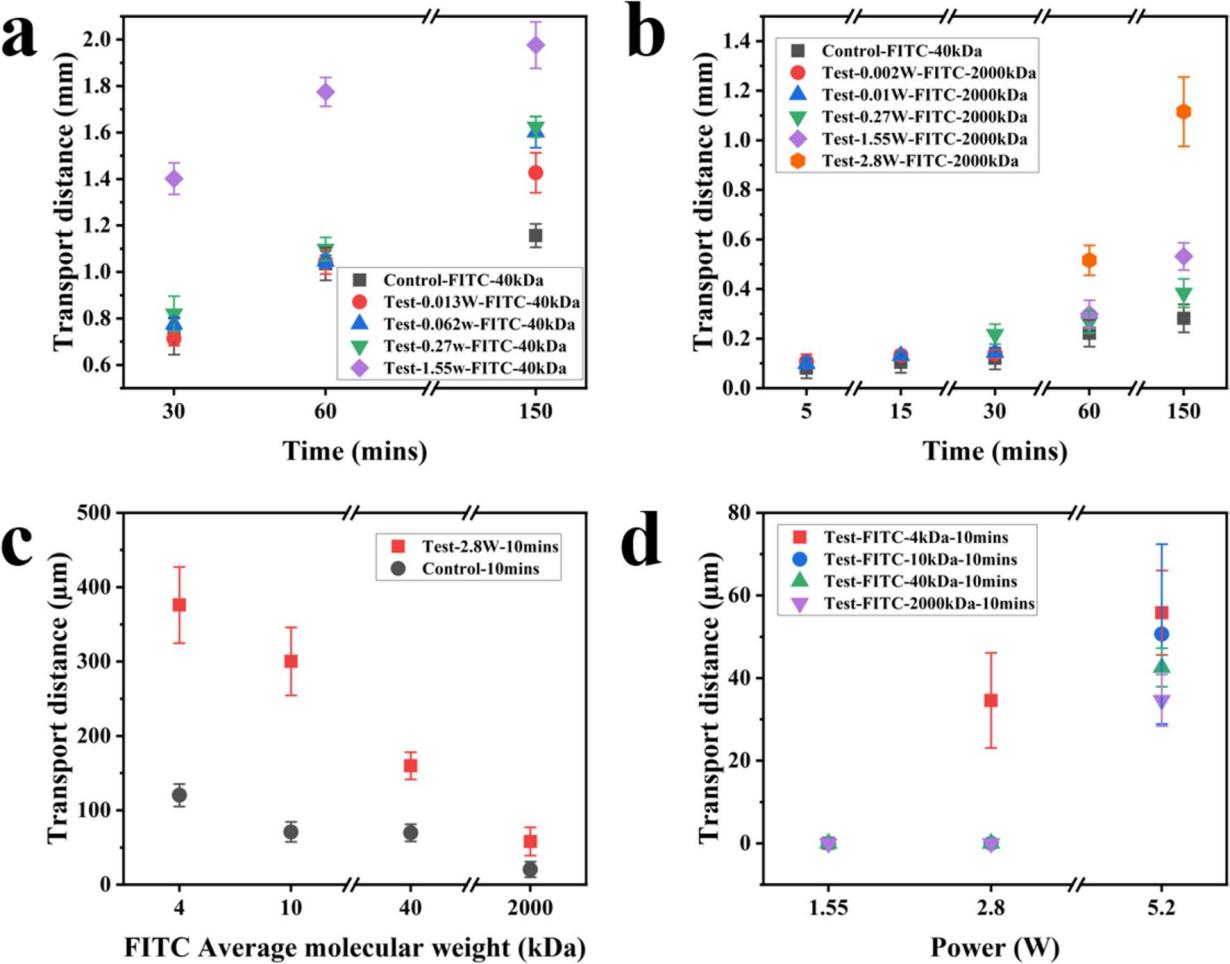
(**a**) Transportation distance of 40 kDa FITC (0.01 mg/ml) into 10% agarose gel under the agitation of SAWs; (**b**) Transportation distance of 2000 kDa FITC (0.01 mg/ml) into 10% agarose gel under the agitation of SAWs; (**c**) Transportation distances of 4 kDa, 10 kDa, 40 kDa, and 2000 kDa FITC (0.01 mg/ml) into 10% agarose gel under the agitation of SAWs in 10 min; (**d**) Transportation distances of 4 kDa, 10 kDa, 40 kDa, and 2000 kDa FITC (0.01 mg/ml) into 20% agarose gel under the agitation of SAWs in 10 min

Ultrasonic gels mixed with FITC of different molecular weights were used for drug diffusion studies at different durations and powers of the SAW agitations, and the obtained results are shown in Fig. 2a. The fluorescence depth images are shown in the supporting information, Figure S4. For the 40 kDa drug molecules, the SAW testing groups all show different degrees of enhancement in transportation depths, as compared to those of the control groups. Molecular transportation distances have achieved their maximum values with the applied SAW power of 1.55 W, and the obtained average transportation distances are approximately 1.40 mm, 1.80 mm and 1.90 mm after agitated by SAW device for 0.5 h, 1 h and 2.5 h, respectively. When the SAW device is excited at a higher power up to 1.55 W, the macromolecules on the surface of the agarose gel are signficantly agitated and become quite mobile. Also, the increased SAW vibrations along the longitudinal direction effectively enhance the drug delivery effect. During the SAW agitation processes, the measured surface temperature on the gel is about 25 to 28℃, which is a slight increase as compared to the room temperature of 22 ℃.

The drug transportation depth results for 2000 kDa FITC are shown in Fig. 2b. The detailed fluoresence microscope images are shown in the supporting informaiton, Figure S5, and Figure S6. As the SAW power and duration are increased, the transportation distances are increased. Compared with those of the 40 kDa experiment results, the transportation distance of the 2000 kDa FITC molecules is signifcantly less, showing that the delivery becomes very difficult. A typical example is that under the same conditions of 1 h and a power of 1.55 W, the transportation distance of the 2000 kDa drug is ~ 0.31 mm, whereas that of the 40 kDa drug is ~ 1.79 mm. For the 2000 kDa drug SAW device experiments, all the experimental groups show significant increases in the transportation distances as compared to that of the control group, even in short experiments such as 5 or 10 min. At the same time, as the power is increased, the transportation distance is also increased. The increase is smaller in the power range of 0.002 W to 1.55 W as compared with those of the 40 kDa molecules testing. When the power is futher increased to 2.8 W, the 1-h testing result shows that the transportation distance of the experimental group is ~ 2.5 times of that of the control group. For the 2.5 h tests, when the input power is 1.55 W, the transportation distance is increased by 0.25 mm as compared with that of the control group. When the input power is 2.8 W, the transportation depth has been increased up to ~ 0.8 mm compared to the control group.

To determine the influence of SAW agitation on transportation depths of various molecular weights, we obtained the correponding results of FITCs with different molecules weights at an input power is 2.8 W, and the results are shown in Fig. 2c. As the average molecular weight of FITC is increased, the transportation distance of both the control and test groups are decreased. Among them, the 4 kDa FITC test results show that the transportation distance of the test group is about 3 times of that of the control group. Whereas for 10 kDa and 40 kDa molecule weights, the control groups all show similar transportation distances. However, the testing groups using the SAWs all show the increased transportation distances by 4.2 and 2.3 times, respectively. At the same time, for the largest molecular weight of 2000 kDa in this study, the testing group shows a significant increase in the molecule delivery distance with a transportation distance of ~ 58 µm, much larger than that of the control group (less than ~ 20 µm). These results clearly show that the SAW device significantly enhance the transportation of large molecules into the agarose gel.

#### Agarose gel density effect

We further compared the delivery results using the agarose gel samples with different concentrations or densities. Figure 2d shows the testing results obtained at different input powers applied onto 20% agarose gel sample. For the 20% agarose gel, due to the reduced water content, the gel block is much harder/denser than 10% ones, and it is difficult for drug molecules to transport into the gels. When the input power is 1.55 W, no FITC diffusion can be observed within 10 min. When the input power is increased to 2.8 W, only the 4 kDa FITC tests show the delivery depth of ~ 34.59 µm, which is about 10 times smaller than that of 10% agarose gel under the same conditions. When the input power is 5.2 W, all different samples with molecular weights of FITCs have shown enhanced transportation distances, but the delivery distance is decreased with the increase of molecular weight.

#### Thermal effects from FSAWs

**Fig. 3:**
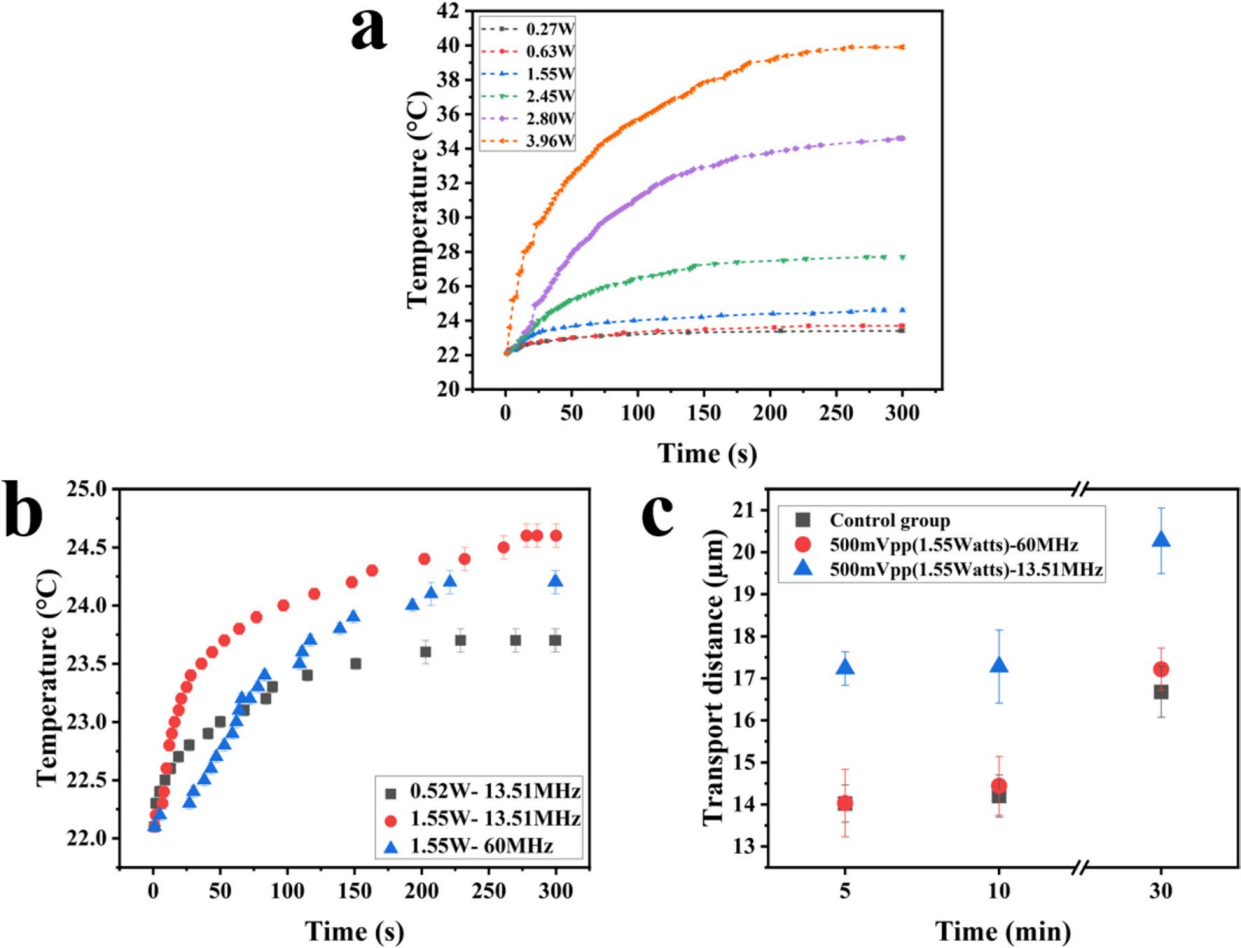
(**a**) The temperature of the SAW device surface changes with time under different applied RF powers; (**b**) The temperature of the SAW device surface changes with time under different frequencie; (**c**) The transportation distance changes with time at different frequencies of 13.5 MHz and 60.0 MHz

To evaluate the thermal effects of the SAW device under room temperature of 22 °C, the temperature changes on the surface of the SAW device were measured at different input RF powers, and the results are shown in Fig. 3a. When the input RF power is 2.8 W, the device’s surface temperature after five minutes is ~ 34.6 °C, which is close to the average body temperature. When the input RF power is 3.96 W, the measured device temperature after five minutes is as high as ~ 39.9 °C. When the input RF power is between 0.2 and 1.55 W, the maximum temperature of the surface of the SAW device does not exceed 25 °C after five minutes. To avoid apparent thermal effect of temperature on transdermal transportation above body temperature, SAW powers of 1.55 W and 2.8 W have been used in the subsequent experiments.

To study the influences of temperature on SAW agitation effect, the signal generator’s input frequency was set as 60.0 MHz (which was far from the resonant frequencies of 13.51 MHz and should not be generating any resonant waves) and the same experiments were performed. Figure 3b shows the changes in the surface temperatures of the SAW device in 300 s under two different frequencies. When the input power is 1.55 W, the surface temperature is 24.2 °C for the 60 MHz test after 300 s, which is ~ 0.4 °C lower than that of the test group of 13.51 MHz. The effect of the signal frequency applied by the FSAW device on transportation distances was further explored, and Fig. 3c shows the variations of results with durations up to 30 min. The transportation distance of the testing group using 60 MHz signal is similar to that of the control group, which is significantly lower than that of the test group using 13.51 MHz at the same power. Results prove that the drug delivery is dominantly caused by acoustic wave agitations, not by the thermal effects in this study.

#### Quantitative transportation characterisation via Franz cells

**Fig. 4:**
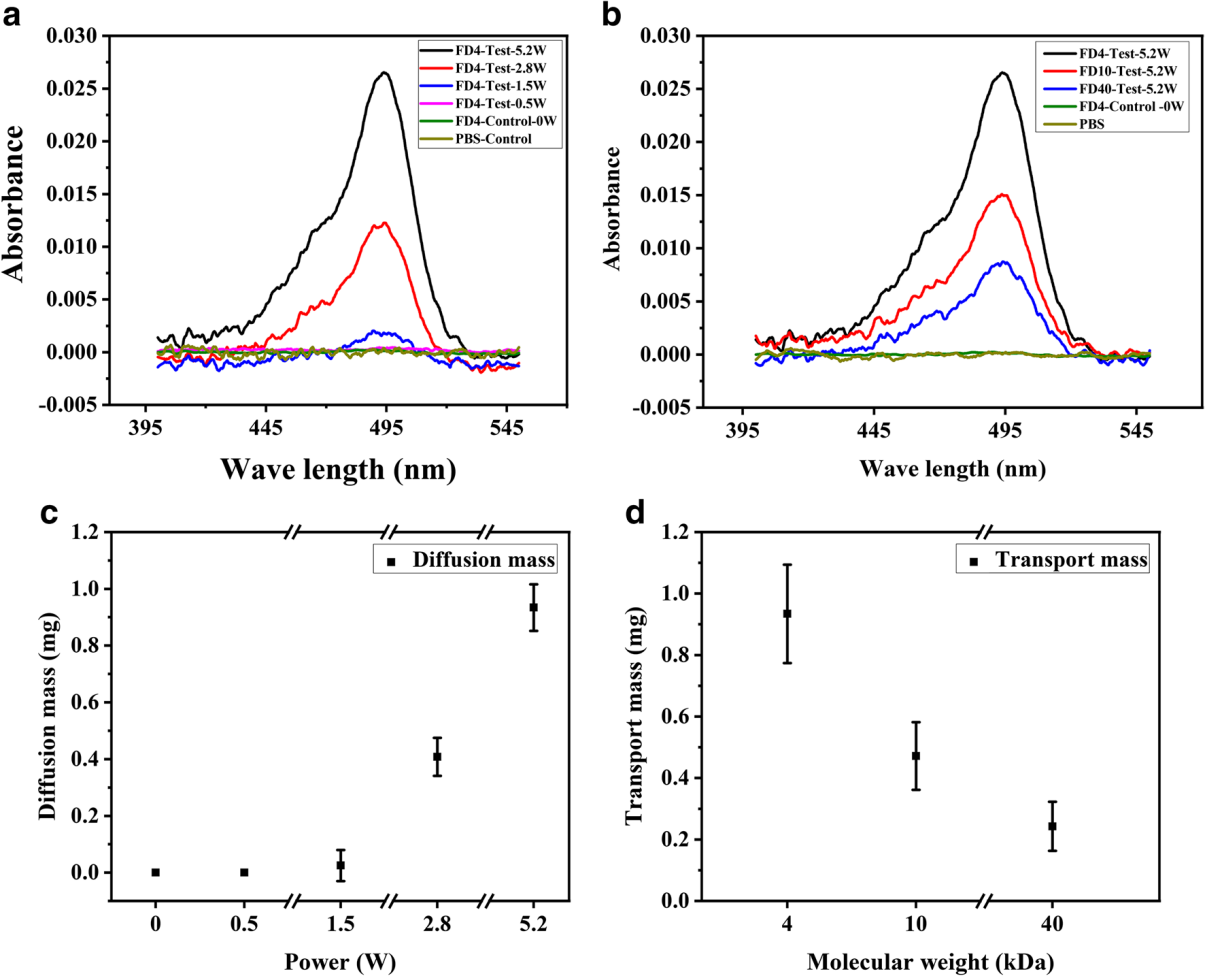
(**a**) Spectra of FITC transportation results in agarose gel tests at different powers within 30 min; (**b**) Spectra of FITC transportation results in agarose gel tests at 5.2 W for different FITC molecules; (**c**) FITC transportation mass in agarose gel tested with different RF power in 30 min; (**d**) FITC transportation mass in the agarose gel tests for different FITC molecules at a power of 5.2 W

Using the Franz cell testing, influences of power variations were studied from both the spectrographic profiles and dynamic diffusion depths of FITC delivered into the agarose gels. The FITC has its maximum absorption peak at 495 nm in the UV–Vis spectrum. Figure 4a shows the solution’s spectral absorption patterns collected after 4 kDa FITC delivery results at different powers in the 30 min tests. Among them, the absorption value reaches the maximum value when the power is 5.2 W. Based on the concentration calibration results and data calculation from the spectra (see Figures S2 and S3 in the supporting information), the corresponding relationship between the power and the total transported mass of FITC in the collection solution was obtained and the results are shown in Fig. 4c. The ratio of the total mass to the total FITC content of 3 mg applied to the agarose gel surface was applied as the percentage of transportation capacity, and the obtained data are summarised in Table S1 in the supporting information. Each set of experiments was repeated three times under the same conditions. For the control group, the transportation mass is almost zero (0.301 ng), which is consistent with the results of the PBS control (0.230 ng). As the power is increased to 0.5 W, the transportation mass is still negligible (~ 0.278 ng). At a power of 1.5 W, the transportation capability is just below 1%. When the power is increased to 2.8 W, the transportation ratio is increased up to ~ 13.6%. As an extreme case, when the power is 5.2 W, the transportation ratio is significantly increased to ~ 31.13%. The enhancing effect of SAW agitation on drug delivery is increased with the applied power, which is consistent with the depth results observed by fluorescence microscopy.

Figure 4b and d depict the delivery characteristics of FITCs with different molecular weights (i.e., 4, 10, and 40 kDa) under a constant power of 5.2 W over 30 min, which highlights the effects of FITC dextran's molecular weight on the drug delivery process. The transportation capacities of 10 kDa and 40 kDa molecule weights become decreased as the molecular weight is increased, i.e., 15.71% and 8.10% respectively. Table S2 in the supporting information summarises the data obtained from Fig. 4b and d, revealing that the higher level of FITC delivery can be achieved with the smaller molecular weight (4 kDa) of FITC molecules.

### Drug diffusion in skin tissues

#### Drug transportation on skin tissue using cryo-sectioning technique

**Fig. 5:**
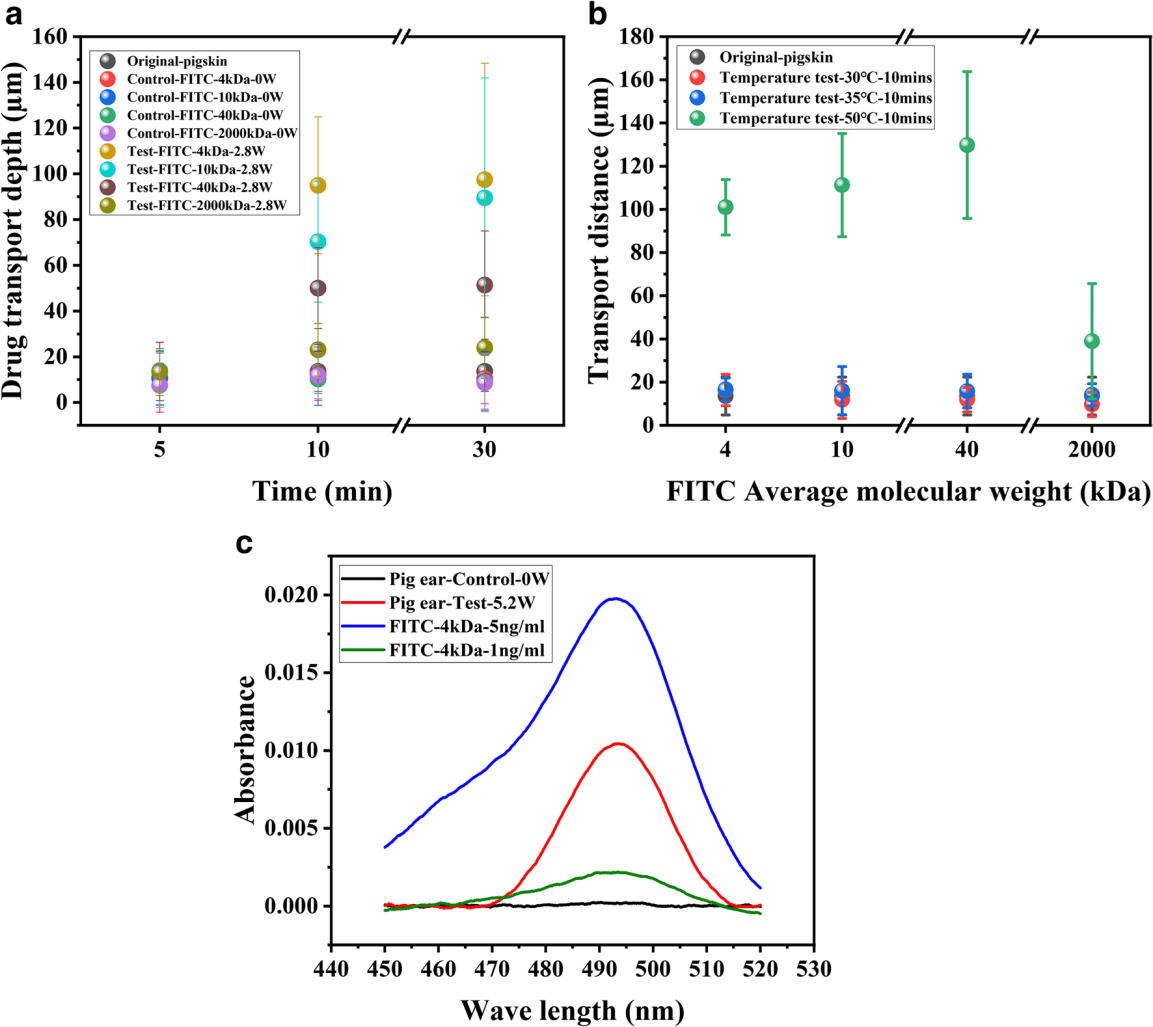
(**a**) Transportation distances of 4 kDa, 10 kDa, 40 kDa, and 2000 kDa FITC (0.5 mg/mL) into pig skin under the agitation of SAW (14.6 MHz); (**b**) Transportation distances of 4 kDa, 10 k Da, 40 kDa, and 2000 kDa FITC (0.5 mg/mL) into pig skin under 30 ℃,35 ℃, and 50 ℃; (**c**) FITC transportation in Pig ear testing at 5.2W

To verify the ability of the drug to diffuse on the skin surface, fresh pig belly was used for the drug diffusion experiment, and the obtained results are shown in Fig. 5a. Results showed that with the increase of time, the transportation distance of the test group using SAW devices is significantly increased. For example, when the molecular weight of FITC is 4 kDa, the transportation depth after 5 min under a power of 2.8 W is ~ 20 µm, whereas the value after 30 min becomes about 100 µm under the same conditions. In addition, the distance is also slightly increased with the applied power for the 2000KDa FITC molecules, e.g., 17 µm for those without applying SAWs, and 22 µm at a SAW power of 2.8 W.

#### Thermal effects on SAW enhanced drug diffusion

To verify the influence of thermal effect on SAW diffusion, a heat plate was used to heat the SAW device which was pressed onto the skin surface without any SAW power input, and the obtained results of temperature changes are shown in Fig. 5b. At a substrate setting temperatures of 30 ℃ and 35 ℃, the maximum transportation distances of 4 kDa FITC are 16.36 and 16.59 microns. However, at a set temperature of ~ 50 ℃, the transportation distance of 4 kDa FITC becomes ~ 100 microns. It shows that the increased temperature has a significant influence on FITC diffusion. However, the temperature was monitored and controlled carefully for all the tests in this study, as it is well-known that this would cause the damage of skin structures. The power applied was quite low (less than 2.8 W) in the SAW-actuated drug delivery, the temperature increase is not significant and this effect on FITC diffusion is rather limited. The testing temperature has not been above 35 ℃, and most of the delivery results are not influenced significantly by such the thermal effects and should be mainly due to agitation of the SAW device which drove the large macromolecule into the skin layer.

#### Drug transportation on skin tissue skin using Franz cell

Figure 5c shows the spectral absorption results of pig ears skin after agitation of 15 min using the FSAW device, along with those of the control group. It takes a certain amount of time for the FITC to be transdermally transmitted into the chamber of the Franz cell. Therefore, after the 15-min SAW induced delivery experiment, the tissue of pig ear was left for one night (~ 19 h) to allow the transported FITC molecules to be continuously diffuse into the chamber of Franz cell. For the control group, no significant signal (below 0.001) was observed at the wavelength of 495 nm. Whereas the testing group has shown an obvious absorption peak around 495 nm, indicating that applying the FSAW effectively promotes the transdermal transportation of FITC. Table 1 summarizes the transportation ratios of the control group and the experimental group, in which that for the experimental group (~ 17.69%) is much higher than the control group one (~ 1.53%). Results prove that the SAW device successfully delivered 4 kDa FITC through the SC and into the skin within 15 min, and a significant absorption peak was observed after 19 h of diffusion process. This provides further evidence that SAW promotes the transportation of macromolecules through the SC and deliver drugs across the skin.

**Table 1:** FITC transportation ratio in Pig ear test at 5.2W

	Transport mass(ng)	Transportation ratio
Pigear-Test-5.2W	530.686	17.69%
Pigear-Control-5.2W	45.977	1.53%

### Discussions on mechanisms of drug delivery

The above experimental results proved the effectiveness of SAW devices in promoting the drug transdermal drug delivery. FSAW devices has driven small molecules such as 4 kDa FITC to successfully diffuse in skin tissue. Based on all the above results, the mechanisms for SAW induced drug transdermal delivery are summarised as following:

1. The high frequency nanoscale vibration and agitation effects with the increased SA powers enhanced the drug delivery capabilities through skin [26, 44]. This mechanism involves direct stimulation by the acoustic waves, under the large SAW induced localised stresses across the skin's surface in both lateral and longitudinal directions. The longitudinal transportation of acoustic waves in skin causes a change in the density of the skin, resulting in cyclic stresses and associated microstructure defects. Therefore, the lipid bilayer structure in SC will be changed and its permeability of drug is increased under the action of localized stresses [45]. The ability of drug transdermal delivery for such effects are dependent on the comprehensive influences of acoustic wave frequency, intensity, exposure area, action time and temperature.
2. Thermal effect accompanying the SAWs affects the skin's permeability of drug molecules. During the activation of SAWs, the thermal effect is generated by energy dissipation on the skin surface. Increase of skin temperature causes the skin to release water, increases the moisture content of the SC, and causes the structure between keratinocytes to become expanded, and creates the gaps [46], thus increasing the skin diffusivity. Temperature also affects the main structural proteins in the SC called keratin, and thus increase the permeability of the SC, promoting the penetration of drug molecules through the skin [47].

There is evidence that thermal effects have limited effects on transdermal transportation [48], which was further verified in this study. The thermal effect of such FSAW can be controlled according to the input energy to avoid thermal damage and promote drug diffusion. The heat dissipation mainly occurs within one or few of wavelength’s depth from the surface, which is much smaller if compared with those of ultrasonic devices used for transdermal transportation. For example, the SAW device with a frequency of 13.51 MHz has a wavelength of 200 microns. Whereas an ultrasonic transducer which has a frequency of 220 kHz has a penetration depth of a few millimeters [49], and related thermal effect is much deeper, and damage could be much higher at a high power.

(1)(3) There are possible generation and oscillation of nanoscale or microscale bubbles on the skin as the SAW devices have resonant frequency in a few MHz [19, 50–53]. The first one is that Rayleigh waves generated by the FSAWs propagate to the solid–liquid interfaces (i.e., between the skin and gel). The other is that the acoustic wave energy is transmitted into the skin surface and encounters microbubbles present in the SC or hair follicles [19, 51]. It is possible that inertial cavitation is commonly generated, and rupture of bubbles causes violent oscillations, which promotes the drug transdermal delivery [52, 53].

Although the drug delivery enhancement effects by the SAWs were verified in this study, it should be imperative to acknowledge various potential sources of variability that could influence these outcomes. The key factors include the age of the porcine specimen, storage conditions, and the inherent thickness of the skin sample. Additional factors affecting permeability rates may include the anatomical location from which the skin was harvested, the animal's age, the freshness of the tissue, and the skin storage parameters. Therefore, the real applications of the SAW devices for the practical application would need to consider a lot of these issues.

## Conclusions

In this study, the FSAW device was successfully demonstrated for drug transdermal delivery and achieved transdermal delivery of macromolecular hydrophilic FITC molecules into pig skin. The FSAW device was more suitable for the practical applications of drug transdermal delivery because it could maintain good performance under bending conditions. In addition, the device can also be integrated as a drug patch, as a wearable device and develop an intelligent driver for time-sharing or timing-driven drug release, which is suitable for precise drug dose delivery control. Results derived from the various experiments have shown that the combined effects of wave frequency and intensity, duration of applied acoustic waves, temperature, and drug molecular weights influence SAW-based transdermal drug delivery. The mechanisms of drug delivery with SAWs were attributed to the effects of nanoscale vibrations and acoustothermal effects induced by SAW during the drug delivery process. Our study highlights the potential of FSAW technology as an efficient method for transdermal delivering large macromolecular drugs. This SAW technology offers a non-invasive alternative compared to conventional drug delivery methods, which often face challenges with large molecule administrations. Future research should be focused on different types of drug molecules and optimised conditions, wearable devices for continuous and controlled drug delivery, and the management of drug delivery for chronic diseases.

## Supplementary Information

Below is the link to the electronic supplementary material.

Supplementary file1 (DOCX 2183 KB)

## Data Availability

All data generated during this study are included in this published article and its supplementary information files.
