# Supplementary material for: Flexible surface acoustic wave technology for enhancing transdermal drug delivery
Source: Drug Deliv Transl Res. 2024 Aug 6;15(4):1363–75. doi: 10.1007/s13346-024-01682-y (PMC11870993; doi:10.1007/s13346-024-01682-y)
Supplement: Supplementary file 1 — Supplementary file1 (DOCX 2183 KB) [file 13346_2024_1682_MOESM1_ESM.docx]

**Support information for paper**

**Flexible surface acoustic wave technology for enhancing transdermal drug delivery**

Jikai Zhang^1^, Duygu Bahar^1^, Hui Ling Ong^1^, Peter Arnold^1^, Meng Zhang^2^, Yunhong Jiang^2^, Ran Tao^3^, Luke Haworth^1^, Xin Yang^4^, Chelsea Brain^5^, Mohammad Rahmati^1^, Hamdi Torun^1^, Qiang Wu,^1^ Jingting Luo,^3^ Yong-Qing Fu^1,^ *

1. Faculty of Engineering and Environment, Northumbria University, Newcastle Upon Tyne, Newcastle NE1 8ST, United Kingdom
2. Hub for Biotechnology in the Built Environment, Department of Applied Sciences, Faculty of Health and Life Sciences, Northumbria University at Newcastle, NE1 8ST, UK
3. Shenzhen Key Laboratory of Advanced Thin Films and Applications, College of Physics and Energy, Shenzhen University, 518060 Shenzhen, China
4. Department of Electrical and Electronic Engineering, School of Engineering, Cardiff University, Cardiff, CF24 3AA, United Kingdom
5. IP & Commercialisation, Research and Innovation, Northumbria University, Newcastle Upon Tyne, Newcastle NE1 8ST, United Kingdom

* Corresponding Author, Prof. Richard Yongqing Fu, email: [Richard.fu@northumbria.ac.uk](mailto:Richard.fu@northumbria.ac.uk)

**Supporting information documents:**

S1. Fabrication of thin film SAW device

S1.1 ZnO thin film deposition

ZnO thin film of about 5 microns was deposited onto aluminum sheets (with a thickness of 200 µm) utilizing a direct current (DC) magnetron sputter (NS3750, Nordiko). A zinc target with a purity of 99.99% was used. During film deposition, the substrate was positioned at 20 cm away from the target, and mixed gases of argon and oxygen with an Ar/O_2_ flow ratio of 10/20 SCCM were used. The DC plasma power was set at 350 Watts, and the chamber pressure was controlled to maintain at ~ 3.75 mTorr. X-ray diffraction (XRD, Siemens D5000 with Cu-Kα radiation, 40 kV, 30 mA) analysis showed that a sharp peak of ZnO (0002) indicating good crystallinity of the ZnO film.

S1.2 Patterning of IDTs

The IDTs were patterned on the surface of the ZnO thin film using conventional microfabrication processes including photolithography and lift-off. A bilayer of Cr/Au was deposited to form the IDTs, with their thicknesses of 20 nm/100 nm, using a thermal evaporator (Edwards AUTO306). The IDTs were designed with a wavelength of 200 µm, comprising 30 pairs of electrodes. The reflection spectra (S11) of the SAW devices were measured using a network analyzer (Keysight, Field Fox N9913A). The resonant frequency (f_0_) of the SAW devices was determined to be 13.51 MHz, and this device generated a mixture of Rayleigh and Lamb waves [1], which will be used for transdermal drug delivery.

Reference:

1. Tao R, Wang WB, Luo JT, Hasan SA, Torun H, Canyelles-Pericas P, ... & Fu YQ. Thin film flexible/bendable acoustic wave devices: Evolution, hybridization and decoupling of multiple acoustic wave modes. Surf Coat Int. 2019; 357, 587-594. https://doi.org/10.1016/j.surfcoat.2018.10.042


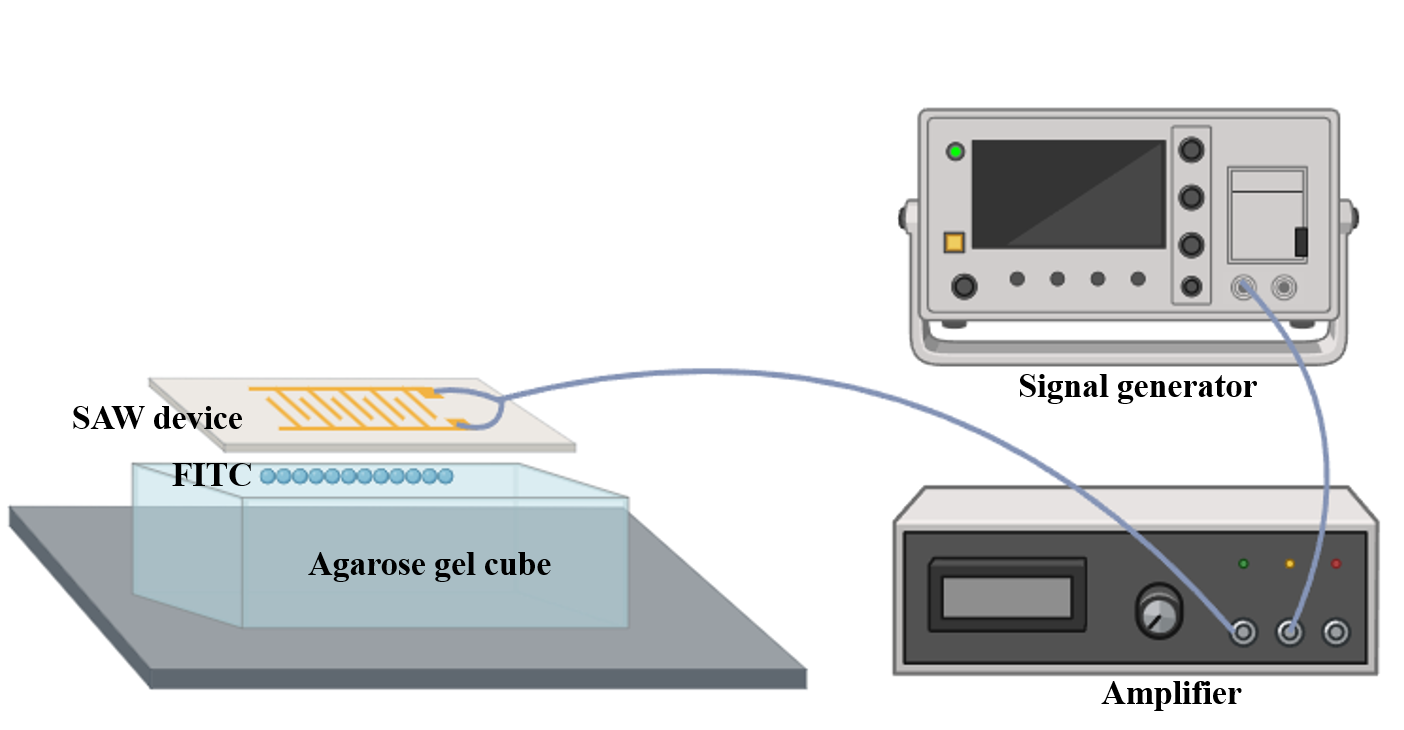


**a**

**b**


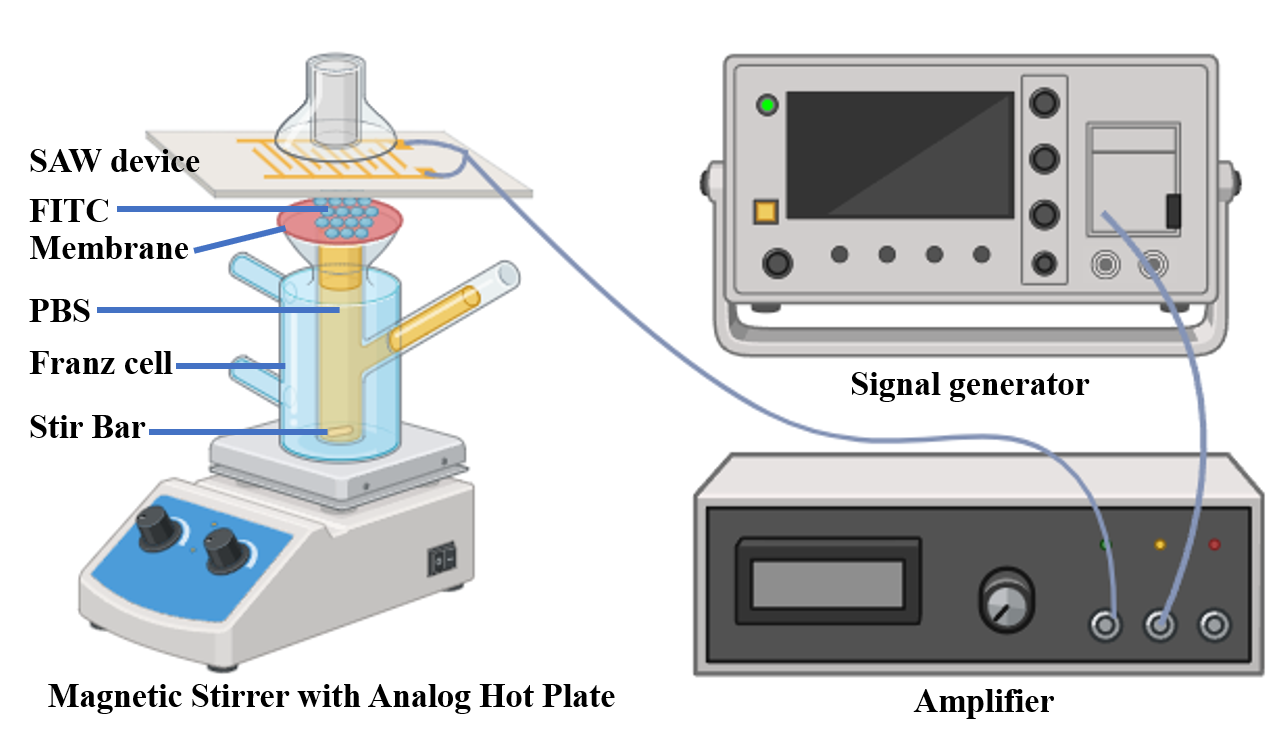


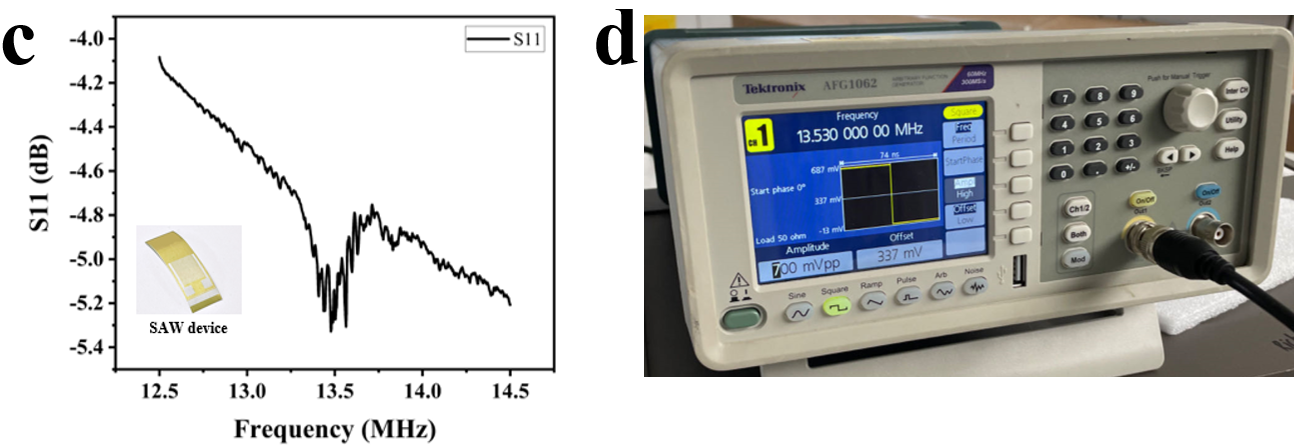


Figure S1. (a) The experiment setup; (b) Franz cell setup; (c) Reflection spectrum (S11) of resonant frequency for the SAW device; (d) Signal generator in setup


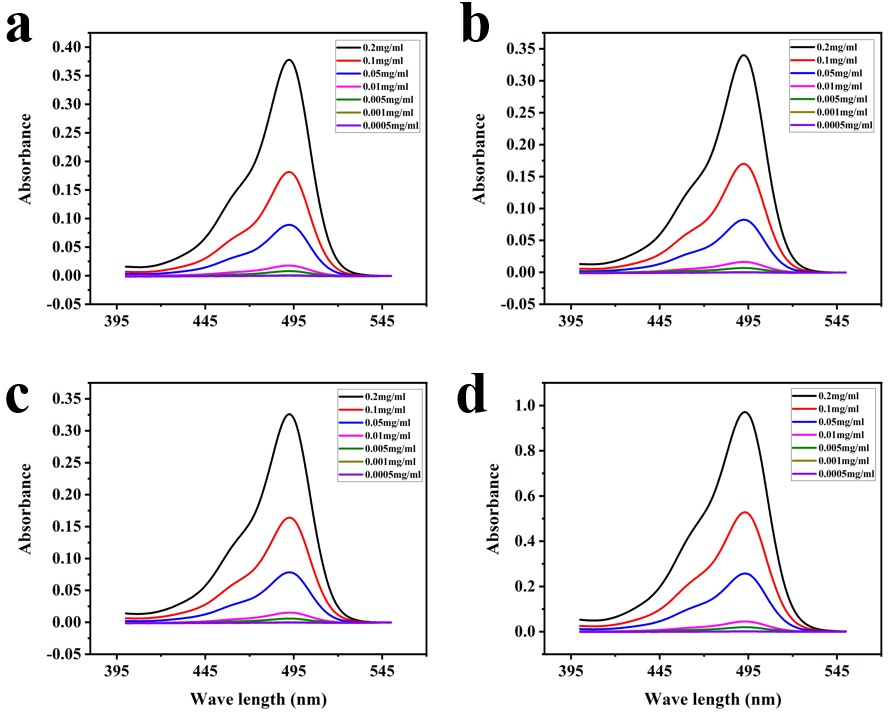


Figure S2. FITC absorbance curves from UV-Vis measurements at different concentrations: (a) 4 kDa, (b)10 kDa, (c) 40 kDa, and (d) 2000 kDa


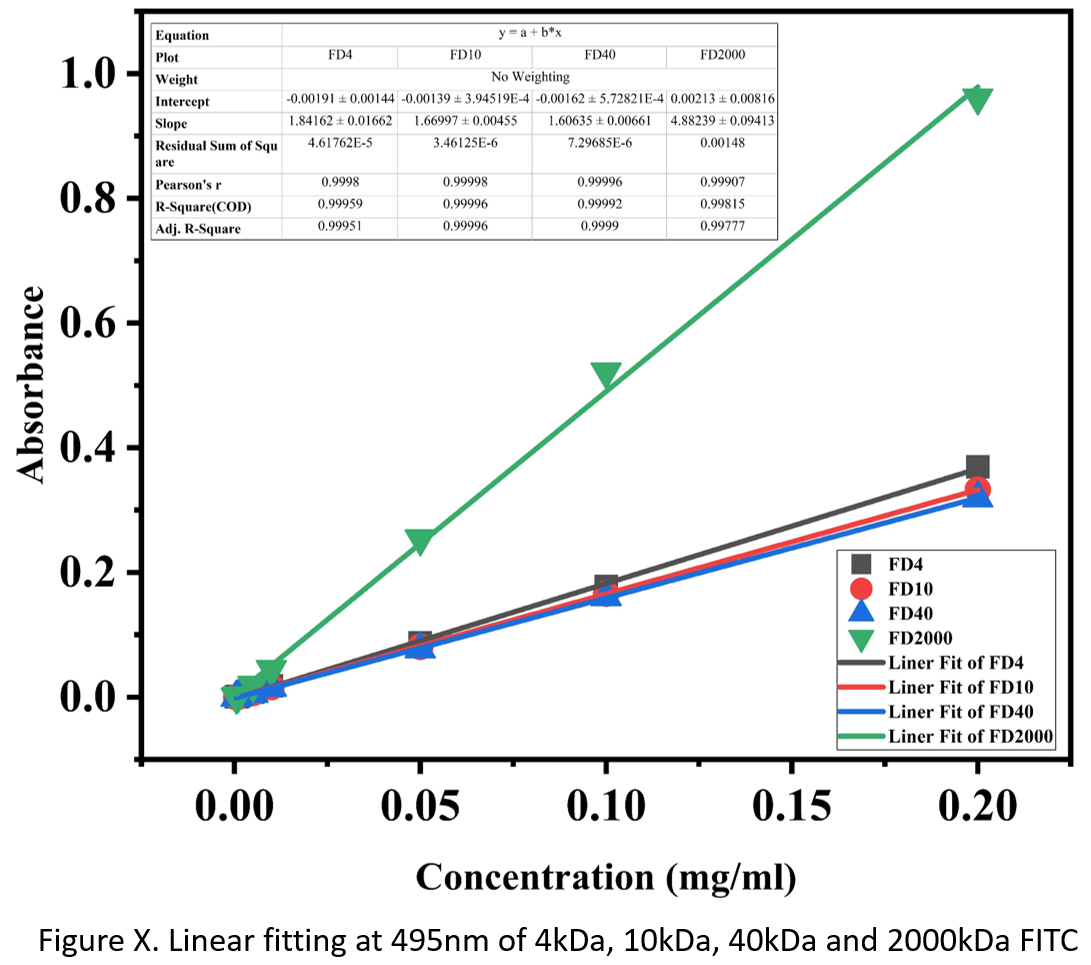


Figure S3. Linear fitting of absorbance spectrum as a function of concentration at a wavelength of 495 nm for 4 kDa, 10 kDa, 40 kDa, and 2000 kDa FITC


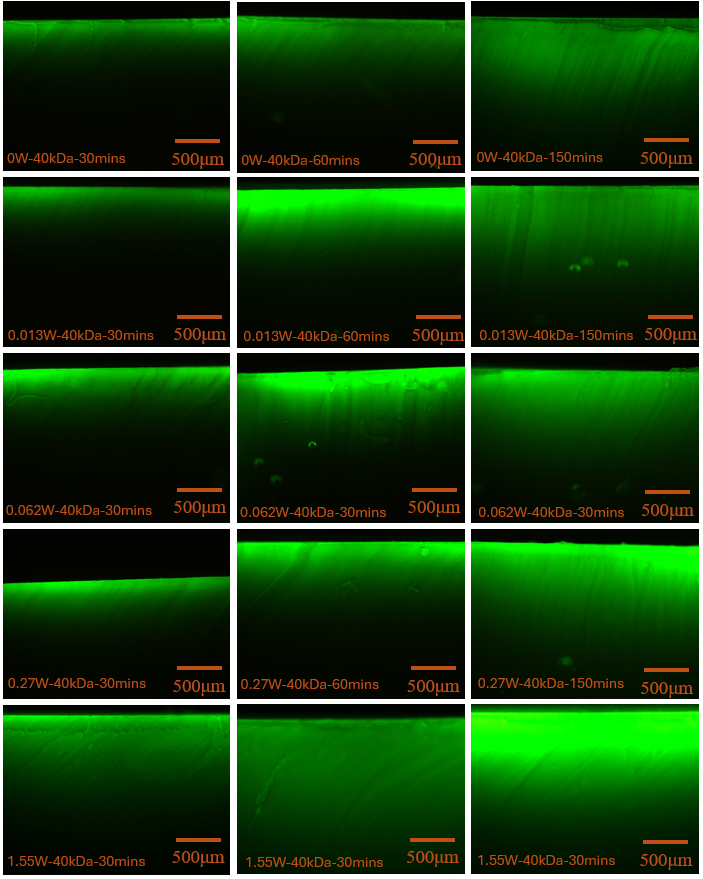


Figure S4. The fluorescence depth images with 40kda FITC at different powers and different times.


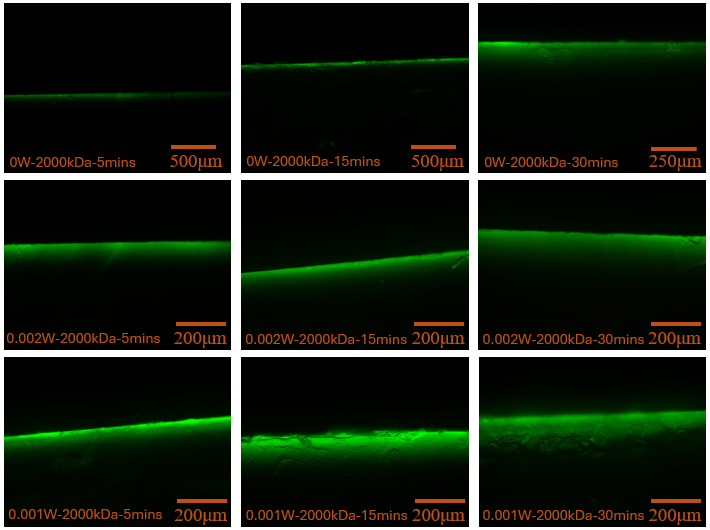


Figure S5. The fluorescence depth images with 2000kda FITC at different powers and different times.


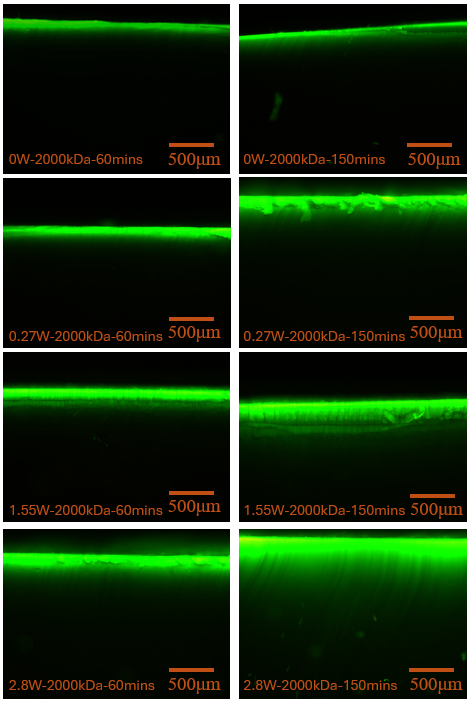


Figure S6. The fluorescence depth images with 2000kda FITC at different powers and different times.

Tabe Sl. FITC transmission ratios in agarose gel test with varied powers in 30 mins

|  | Diffusion mass(mg) | Transmission ratio |
| --- | --- | --- |
| FD4-Test-5.2W | 0.933996 | 31.13% |
| FD4-Test-2.8W | 0.408036 | 13.60% |
| FD4-Test-1.5W | 0.024983 | 0.83% |
| FD4-Test-0.5W | 0.000301 | 0.01% |
| FD4-Control-0W | 0.000230 | 0.01% |
| PBS-Control | 0.000278 | 0.01% |

Table S2. FITC transmission ratios of different FITC molecules in agarose gel test at an RF power of 5.2 W

|  | Diffusion mass(mg) | Transmission ratio |
| --- | --- | --- |
| FD4-Test-5.2W | 0.933996 | 31.13% |
| FD10-Test-5.2W | 0.471330 | 15.71% |
| FD40-Test-5.2W | 0.242999 | 8.10% |
